# Editorial: Advances in the diagnosis and management of infectious diseases

**DOI:** 10.3389/fcimb.2025.1765521

**Published:** 2026-01-06

**Authors:** Diana Manolescu, Emil Robert Stoicescu, Ariadna Petronela Fildan

**Affiliations:** 1 Radiology and Medical Imaging University Clinic, ‘Victor Babes’ University of Medicine and Pharmacy Timisoara, Timisoara, Romania; 2 Center for Research and Innovation in Precision Medicine of Respiratory Diseases (CRIPMRD), ‘Victor Babes’ University of Medicine and Pharmacy Timisoara, Timisoara, Romania; 3 Research Center for Medical Communication, ‘Victor Babes’ University of Medicine and Pharmacy Timisoara, Timisoara, Romania; 4 Research Center for Pharmaco-Toxicological Evaluations, ‘Victor Babes’ University of Medicine and Pharmacy Timisoara, Timisoara, Romania; 5 Faculty of Medicine, “Ovidius” University of Constanta, Constanta, Romania

**Keywords:** antimicrobial resistance (AMR), biomarker discovery, diagnostic stewardship, infectious diseases, molecular sequencing, point-of-care testing, precision medicine

## Introduction

Infectious diseases continue to represent one of the most dynamic and rapidly evolving domains of modern medicine. Global interconnectedness, climate change, and microbial adaptation have contributed to the continuous emergence and re-emergence of pathogens, while the parallel rise of antimicrobial resistance (AMR) challenges conventional therapeutic paradigms. Against this complex backdrop, diagnostic innovation has become the cornerstone of effective clinical and public-health response. Advances in molecular detection, sequencing technologies, point-of-care testing, and quantitative biomarker analysis are redefining how clinicians detect, characterize, and monitor infectious processes.

The present Research Topic, *“Advances in the Diagnosis and Management of Infectious Diseases,”* compiles twenty peer-reviewed studies that collectively capture the ongoing transition from laboratory innovation to clinical translation. The selected papers span a broad scientific and geographical spectrum, encompassing next-generation sequencing (NGS), chemiluminescent and immunoassay technologies, imaging biomarkers, antimicrobial stewardship strategies, and outcome-oriented clinical research. Taken together, these studies exemplify a global research effort directed toward improving diagnostic accuracy, therapeutic precision, and patient prognosis through technological convergence and multidisciplinary collaboration.

To synthesize this body of work, the articles were organized into five major research directions:

(1) Innovations in Molecular and Sequencing-Based Diagnostics;
(2) Clinical Impact of Metagenomic Sequencing in Infectious Diseases;
(3) Development of Rapid and Point-of-Care Diagnostic Tools;
(4) Diagnostic Performance and Biomarker Discovery; and
(5) Antimicrobial Resistance, Stewardship, and Clinical Outcomes.

Each direction reflects a distinct yet interconnected facet of the current diagnostic landscape and collectively illustrates how precision technology and clinical integration are reshaping infectious disease management worldwide.

## Innovations in molecular and sequencing-based diagnostics

The first cluster of studies underscores the growing maturity of molecular and sequencing-based platforms capable of delivering comprehensive pathogen detection with clinical-grade accuracy. Technologies such as third-generation nanopore sequencing, targeted NGS (tNGS), and metagenomic NGS (mNGS) now offer near-real-time identification of microorganisms and resistance determinants directly from clinical specimens, reducing dependence on culture and expediting targeted therapy.

A large retrospective study evaluating third-generation nanopore sequencing in non-tuberculous mycobacterial pulmonary disease (NTMPD) demonstrated 81.3% sensitivity and 98.8% specificity, outperforming culture and identifying numerous low-burden or mixed infections undetected by conventional methods. Similarly, validation of the MeltPlus TB-NTM/RIF assay confirmed its capacity to simultaneously identify *Mycobacterium tuberculosis* complex, non-tuberculous mycobacteria, and rifampicin resistance with high accuracy within three hours—an achievement of substantial operational relevance for tuberculosis-endemic settings (Zhao et al., Wang et al.).

Complementing these advances, a multiplex molecular point-of-care platform (fastNTM) achieved near-perfect agreement with the Xpert MTB/RIF Ultra assay, reducing total diagnostic time to 90 minutes and demonstrating the feasibility of decentralizing complex molecular testing. Parallel studies comparing tNGS and mNGS in respiratory infections revealed equivalent diagnostic performance but lower cost and faster turnaround for tNGS, emphasizing the importance of targeted panels as cost-effective alternatives to broad metagenomic approaches. In fungal respiratory infections, both modalities far outperformed culture, with *Pneumocystis jirovecii*, *Candida albicans*, and *Aspergillus fumigatus* emerging as the most prevalent organisms (Yi et al., Kuang et al.).

Collectively, these studies illustrate the ongoing convergence between sequencing depth, diagnostic speed, and cost-efficiency. The data support an impending shift from exploratory sequencing toward standardized, clinically integrated molecular workflows, where sequencing informs real-time antimicrobial and infection-control decisions.

## Clinical impact of metagenomic sequencing in infectious diseases

The second group of articles focuses on the clinical translation of metagenomic sequencing in complex or atypical infections, particularly those affecting immunocompromised hosts. The ability of mNGS to detect microbial DNA or RNA directly from plasma, cerebrospinal fluid, or bronchoalveolar lavage fluid (BALF) has transformed the diagnostic outlook for disseminated and opportunistic diseases that are frequently missed by culture.

In a study on disseminated tuberculosis, plasma mNGS detected *Mycobacterium tuberculosis* in two-thirds of confirmed cases, achieving 100% specificity and outperforming traditional immune-based assays, especially in HIV-positive patients (Ma et al.).

Another large retrospective analysis from Wuhan evaluated BALF mNGS in nearly 300 patients with suspected pneumonia and demonstrated a diagnostic sensitivity of 87.9%, significantly higher than that of conventional microbial tests. The inclusion of immunocompromised and oncologic populations emphasized the added value of mNGS in guiding tailored therapy for patients with ambiguous or culture-negative infections (Zhou et al.).

The same principle was applied in non-endemic visceral leishmaniasis, where mNGS successfully identified *Leishmania infantum* and *L. donovani* in peripheral blood and bone marrow samples of previously misdiagnosed patients. Early molecular identification allowed rapid initiation of specific antiprotozoal therapy and complete recovery—highlighting mNGS as a powerful tool for identifying imported or unexpected pathogens (Zhao et al.).

Taken together, these studies demonstrate that the clinical utility of mNGS extends beyond discovery; it meaningfully alters diagnostic certainty, accelerates therapeutic decisions, and supports differential diagnosis in both endemic and non-endemic contexts. However, they also underscore the need for standardized interpretation frameworks and cost-effectiveness analyses before universal adoption.

## Development of rapid and point-of-care diagnostic tools

Rapid, field-deployable diagnostics are indispensable for outbreak response, rural healthcare, and veterinary surveillance. The third research direction presents a suite of isothermal amplification-based assays, often combined with CRISPR/Cas detection systems or lateral flow devices (LFDs), which achieve high analytical performance without sophisticated infrastructure.

A recombinase polymerase amplification (RPA)/LFD assay for *Dengue virus* type 2 detection achieved a limit of detection of approximately 40 copies per reaction within 20 minutes, maintaining stability across variable temperatures and multiple freeze–thaw cycles. This portability renders it particularly valuable for tropical regions where dengue is endemic but laboratory capacity limited (Prescott et al.).

Similarly, a CRISPR-Cas12a-enhanced RPA assay for *goose parvovirus* achieved single-digit copy detection in under an hour, outperforming qPCR in sensitivity and specificity and allowing naked-eye readout under blue light. These designs collectively demonstrate how CRISPR and RPA technologies are merging into flexible, low-cost diagnostic systems adaptable across species and pathogens (Chen et al.).

A third innovation, the immunoassay–mass spectrometry (MS) method for *Brucella melitensis*, integrated magnetic nanoparticle capture with MALDI-TOF identification, eliminating the need for lengthy culture of hazardous organisms. With a detection limit of 50 CFU/mL and completion within 60 minutes, the method markedly improves biosafety and turnaround time for a major zoonotic pathogen (Sharif et al.).

Collectively, these studies reinforce the promise of next-generation point-of-care (POC) platforms: assays that combine analytical sensitivity approaching laboratory methods with operational simplicity suitable for decentralized or emergency settings. Their broad adoption could bridge existing gaps between molecular capability and field applicability, thereby extending the reach of precision diagnostics to low-resource environments.

## Diagnostic performance and biomarker discovery

The fourth group of studies emphasizes quantitative biomarkers and advanced immunoassays that transcend binary diagnostic outcomes by offering dynamic measures of disease burden and therapeutic response. As infectious disease management evolves toward precision medicine, such biomarkers serve as vital adjuncts to guide initiation, monitoring, and discontinuation of therapy.

A multicenter comparison of chemiluminescent immunoassay (CLIA) and lateral flow assay (LFA) for cryptococcal antigen detection revealed near-perfect agreement (99.2%) but highlighted CLIA’s superiority for longitudinal monitoring (Tang et al.).

In musculoskeletal infections, synovial fluid fibrin degradation product (sFDP) emerged as a robust biomarker for periprosthetic joint infection (PJI), showing diagnostic accuracy comparable to CRP and ESR but with greater procedural practicality. The study advocates combined biomarker approaches to refine preoperative evaluation, particularly when culture or histopathology is delayed (Huang et al.).

Meanwhile, analysis of plasma *Epstein–Barr virus* (EBV) DNA load in intestinal EBV infection established its dual diagnostic and prognostic value. Defined thresholds accurately distinguished infection types and predicted six-month outcomes, with viral load correlating closely with tissue EBER positivity. The work supports integration of plasma EBV quantification into monitoring algorithms for EBV-associated intestinal disorders and lymphoproliferative disease (Ma et al.).

These contributions collectively signal a transition from static qualitative tests toward dynamic, data-rich biomarker systems capable of stratifying risk, quantifying disease progression, and informing therapeutic decisions in real time.

## Antimicrobial resistance, stewardship, and clinical outcomes

The final thematic group addresses the intertwined challenges of antimicrobial resistance (AMR), clinical stewardship, and outcome prediction—domains where diagnostic precision directly influences survival and antibiotic sustainability.

A 2023 cohort of 190 patients with carbapenem-resistant *Klebsiella pneumoniae* (CRKP) identified ICU admission, co-infection with CRAB or CRPA, and elevated neutrophil-to-lymphocyte ratio (NLR) as independent predictors of mortality, whereas timely appropriate therapy was protective. The NLR, a simple hematologic parameter, demonstrated moderate predictive accuracy (AUC 0.696) and offers a practical adjunct for early risk stratification in critically ill patients (Wang et al.).

From a systems perspective, a six-year initiative at the Central Texas Veterans Health Care System examined the impact of discontinuing automatic reflex urine cultures after urinalysis. The policy reduced culture volume by 42%, lowered unnecessary antibiotic use, and shifted prescribing patterns toward narrower agents such as nitrofurantoin—an exemplary case of diagnostic stewardship aligning laboratory practice with evidence-based care (Berger et al.).

Two COVID-19-related studies further elucidate the interplay between diagnostics, host factors, and outcomes. A Romanian cohort comparing lopinavir/ritonavir and darunavir/ritonavir demonstrated a survival advantage only for lopinavir-based therapy, highlighting the importance of comparative effectiveness data during therapeutic repurposing. Another investigation during the Omicron wave in China revealed a high incidence (29%) of COVID-19-associated pulmonary aspergillosis (CAPA), with mortality exceeding 50%. Diabetes, chronic lung disease, corticosteroid exposure, and mechanical ventilation emerged as key risk factors, emphasizing the need for routine fungal screening and judicious use of immunomodulators. (Paroczai et al., Xiao et al.).

Additional studies addressed diagnostic limitations and epidemiologic trends. Evaluation of HTLV-1 antibody testing in endemic Chinese hospitals revealed insufficient sensitivity at current cutoff levels, supporting combined serologic and nucleic-acid testing for high-risk cases (Chen et al.).

A regional surveillance study from Shijiazhuang documented post-pandemic resurgence and compositional shifts in acute respiratory infection (ARI) pathogens, with *Mycoplasma pneumoniae* prevalence rising sharply after withdrawal of non-pharmaceutical interventions (Zheng et al.).

Together, these investigations illustrate how accurate diagnostics underpin not only pathogen detection but also the broader ecosystem of stewardship, surveillance, and patient safety. They collectively argue for an integrated approach linking laboratory insight to clinical policy and outcome analysis.

## Future directions and perspectives

The twenty studies represented in this Research Topic converge on a clear trajectory: the integration of multimodal, data-driven, and personalized strategies into infectious disease diagnostics and management. Several cross-cutting trends emerge.

First, the acceleration of molecular turnaround time—from days to minutes—signals an imminent reconfiguration of diagnostic algorithms. As nanopore and RPA-based assays mature, decentralization of advanced molecular testing will become feasible even in resource-limited settings. However, harmonization of analytic performance metrics and quality assurance frameworks remains an essential prerequisite for equitable global adoption.

Second, data integration and artificial intelligence (AI) are poised to transform diagnostic interpretation. Sequencing output, biomarker kinetics, and radiologic patterns can be synthesized through machine-learning models to enable predictive diagnostics, capable of forecasting resistance emergence or treatment response before clinical deterioration occurs. Embedding these models into electronic health record systems will be critical to realizing their full potential.

Third, the field must address economic and operational scalability. Despite demonstrated accuracy, mNGS and other high-throughput platforms remain cost-intensive and technically demanding. Development of streamlined bioinformatic pipelines, cloud-based analysis, and standardized reporting formats will be key to reducing barriers to clinical implementation.

Finally, interdisciplinary collaboration stands as the defining catalyst for sustained progress. The convergence of microbiology, molecular engineering, informatics, imaging, and clinical medicine exemplified in this Research Topic underscores that infectious disease diagnostics can no longer operate in disciplinary isolation. Future research should continue to pursue multicenter validation, longitudinal follow-up, and implementation science to ensure that innovation translates into measurable public-health benefit.

## Conclusion

This Research Topic encapsulates a vibrant and rapidly advancing research landscape in infectious disease diagnostics and management. Collectively, these works demonstrate that the future of infection control and clinical microbiology lies in precision, speed, and integration—precision through genomics and quantitative biomarkers, speed through point-of-care technologies, and integration through stewardship frameworks and digital analytics.

By uniting technological innovation with clinical applicability, the contributions in this Research Topic offer a roadmap toward more personalized, predictive, and preventive infectious disease care. Continued global collaboration, standardization, and equitable dissemination of these advances will be essential to translate diagnostic excellence into improved outcomes for patients worldwide.

